# Supplementary material for: Modeling glioblastoma heterogeneity as a dynamic network of cell states
Source: Mol Syst Biol. 2021 Sep 16;17(9):e10105. doi: 10.15252/msb.202010105 (PMC8444284; doi:10.15252/msb.202010105)
Supplement: Supplementary file 6 — Source Data for Figure 5 [file MSB-17-e10105-s004.zip › Figure5A_sourcedata/GSEA_3017/nosigdb_stateF.GseaPreranked.1622795015644/DOROSZKO_U3180MG_CLUSTER_3.html]

Details for gene set DOROSZKO\_U3180MG\_CLUSTER\_3[GSEA]

|  || Dataset | state33017 |
| Phenotype | NoPhenotypeAvailable |
| Upregulated in class | na\_neg |
| GeneSet | DOROSZKO\_U3180MG\_CLUSTER\_3 |
| Enrichment Score (ES) | -0.78045964 |
| Normalized Enrichment Score (NES) | -6.1262536 |
| Nominal p-value | 0.0 |
| FDR q-value | 0.0 |
| FWER p-Value | 0.0 |
Table: GSEA Results Summary

  

Fig 1: Enrichment plot: DOROSZKO\_U3180MG\_CLUSTER\_3      
 Profile of the Running ES Score & Positions of GeneSet Members on the Rank Ordered List

  

| PROBE | GENE SYMBOL | GENE\_TITLE | RANK IN GENE LIST | RANK METRIC SCORE | RUNNING ES | CORE ENRICHMENT || 1 | DDB2 |  |  | 143 | 0.682 | -0.0690 | No |
| 2 | TCEAL9 |  |  | 146 | 0.673 | -0.0663 | No |
| 3 | IKBIP |  |  | 567 | 0.408 | -0.2775 | No |
| 4 | LRRCC1 |  |  | 690 | 0.374 | -0.3375 | No |
| 5 | ETFB |  |  | 901 | 0.326 | -0.4424 | No |
| 6 | METRN |  |  | 903 | 0.325 | -0.4412 | No |
| 7 | SCARA3 |  |  | 1096 | 0.293 | -0.5372 | No |
| 8 | PIDD1 |  |  | 1218 | 0.277 | -0.5971 | No |
| 9 | PSMB9 |  |  | 1347 | 0.262 | -0.6608 | No |
| 10 | ABHD3 |  |  | 1382 | 0.259 | -0.6766 | No |
| 11 | CSRP2 |  |  | 1386 | 0.258 | -0.6768 | No |
| 12 | RFC5 |  |  | 1473 | -0.250 | -0.7191 | No |
| 13 | RPA2 |  |  | 1490 | -0.256 | -0.7258 | No |
| 14 | CCT2 |  |  | 1494 | -0.258 | -0.7260 | No |
| 15 | NCAPH2 |  |  | 1496 | -0.258 | -0.7251 | No |
| 16 | ITGB1BP1 |  |  | 1501 | -0.259 | -0.7257 | No |
| 17 | ACAT2 |  |  | 1512 | -0.263 | -0.7294 | No |
| 18 | MSH6 |  |  | 1517 | -0.264 | -0.7300 | No |
| 19 | RBBP8 |  |  | 1518 | -0.265 | -0.7285 | No |
| 20 | NUDT5 |  |  | 1522 | -0.265 | -0.7286 | No |
| 21 | MAD2L2 |  |  | 1539 | -0.269 | -0.7353 | No |
| 22 | MCMBP |  |  | 1546 | -0.271 | -0.7369 | No |
| 23 | PSIP1 |  |  | 1564 | -0.275 | -0.7440 | No |
| 24 | PIN1 |  |  | 1565 | -0.275 | -0.7425 | No |
| 25 | WDHD1 |  |  | 1569 | -0.277 | -0.7425 | No |
| 26 | PAXX |  |  | 1575 | -0.279 | -0.7436 | No |
| 27 | PPIH |  |  | 1579 | -0.281 | -0.7436 | No |
| 28 | E2F1 |  |  | 1580 | -0.281 | -0.7421 | No |
| 29 | PRKDC |  |  | 1594 | -0.285 | -0.7471 | No |
| 30 | RCC1 |  |  | 1604 | -0.286 | -0.7501 | No |
| 31 | RAD1 |  |  | 1613 | -0.289 | -0.7526 | No |
| 32 | CENPX |  |  | 1621 | -0.291 | -0.7546 | No |
| 33 | POLA2 |  |  | 1622 | -0.291 | -0.7530 | No |
| 34 | TMEM97 |  |  | 1629 | -0.293 | -0.7545 | No |
| 35 | GINS1 |  |  | 1634 | -0.295 | -0.7549 | No |
| 36 | PARP2 |  |  | 1665 | -0.305 | -0.7685 | No |
| 37 | KLHL23 |  |  | 1674 | -0.308 | -0.7709 | No |
| 38 | CEP57 |  |  | 1676 | -0.309 | -0.7698 | No |
| 39 | RDX |  |  | 1678 | -0.310 | -0.7686 | No |
| 40 | EIF4EBP1 |  |  | 1681 | -0.312 | -0.7679 | No |
| 41 | FADS1 |  |  | 1682 | -0.312 | -0.7662 | No |
| 42 | OIP5 |  |  | 1686 | -0.314 | -0.7660 | No |
| 43 | CTPS1 |  |  | 1692 | -0.316 | -0.7669 | No |
| 44 | MGME1 |  |  | 1697 | -0.317 | -0.7672 | No |
| 45 | PRIM1 |  |  | 1703 | -0.320 | -0.7680 | No |
| 46 | H1FX |  |  | 1705 | -0.321 | -0.7668 | No |
| 47 | HAUS1 |  |  | 1708 | -0.322 | -0.7660 | No |
| 48 | CENPQ |  |  | 1711 | -0.322 | -0.7653 | No |
| 49 | PARP1 |  |  | 1713 | -0.323 | -0.7641 | No |
| 50 | NFYB |  |  | 1721 | -0.326 | -0.7659 | No |
| 51 | RALY |  |  | 1729 | -0.330 | -0.7676 | No |
| 52 | MCM3 |  |  | 1738 | -0.335 | -0.7699 | No |
| 53 | GPN3 |  |  | 1739 | -0.335 | -0.7681 | No |
| 54 | XRN2 |  |  | 1750 | -0.340 | -0.7713 | No |
| 55 | COA1 |  |  | 1753 | -0.341 | -0.7705 | No |
| 56 | GLO1 |  |  | 1755 | -0.342 | -0.7691 | No |
| 57 | SUZ12 |  |  | 1766 | -0.347 | -0.7723 | No |
| 58 | FKBP3 |  |  | 1769 | -0.347 | -0.7715 | No |
| 59 | SLF1 |  |  | 1773 | -0.350 | -0.7711 | No |
| 60 | HNRNPA3 |  |  | 1776 | -0.351 | -0.7702 | No |
| 61 | WDR76 |  |  | 1783 | -0.355 | -0.7713 | No |
| 62 | SRPK1 |  |  | 1788 | -0.356 | -0.7714 | No |
| 63 | TMEM107 |  |  | 1789 | -0.357 | -0.7695 | No |
| 64 | BRIP1 |  |  | 1790 | -0.357 | -0.7676 | No |
| 65 | POC1A |  |  | 1794 | -0.360 | -0.7671 | No |
| 66 | LIG1 |  |  | 1797 | -0.361 | -0.7662 | No |
| 67 | WDR34 |  |  | 1806 | -0.365 | -0.7683 | No |
| 68 | XRCC6 |  |  | 1825 | -0.377 | -0.7754 | No |
| 69 | CDC45 |  |  | 1836 | -0.382 | -0.7784 | Yes |
| 70 | BARD1 |  |  | 1839 | -0.384 | -0.7773 | Yes |
| 71 | CCT4 |  |  | 1840 | -0.384 | -0.7752 | Yes |
| 72 | MASTL |  |  | 1843 | -0.385 | -0.7742 | Yes |
| 73 | FANCA |  |  | 1844 | -0.385 | -0.7721 | Yes |
| 74 | MMS22L |  |  | 1846 | -0.387 | -0.7705 | Yes |
| 75 | SVIP |  |  | 1849 | -0.388 | -0.7694 | Yes |
| 76 | CDCA4 |  |  | 1852 | -0.391 | -0.7683 | Yes |
| 77 | CEP135 |  |  | 1855 | -0.393 | -0.7672 | Yes |
| 78 | ACTL6A |  |  | 1859 | -0.393 | -0.7666 | Yes |
| 79 | E2F8 |  |  | 1864 | -0.396 | -0.7665 | Yes |
| 80 | EXOSC8 |  |  | 1865 | -0.397 | -0.7643 | Yes |
| 81 | PPP1CA |  |  | 1872 | -0.399 | -0.7652 | Yes |
| 82 | PKMYT1 |  |  | 1876 | -0.402 | -0.7646 | Yes |
| 83 | TEDC1 |  |  | 1883 | -0.410 | -0.7654 | Yes |
| 84 | SHCBP1 |  |  | 1886 | -0.412 | -0.7642 | Yes |
| 85 | RFC4 |  |  | 1887 | -0.413 | -0.7619 | Yes |
| 86 | SAE1 |  |  | 1893 | -0.417 | -0.7622 | Yes |
| 87 | HDAC2 |  |  | 1896 | -0.418 | -0.7610 | Yes |
| 88 | ASF1B |  |  | 1897 | -0.419 | -0.7587 | Yes |
| 89 | RAD18 |  |  | 1898 | -0.419 | -0.7564 | Yes |
| 90 | POLE3 |  |  | 1901 | -0.426 | -0.7551 | Yes |
| 91 | MXD3 |  |  | 1905 | -0.428 | -0.7543 | Yes |
| 92 | BAZ1B |  |  | 1910 | -0.429 | -0.7540 | Yes |
| 93 | MNS1 |  |  | 1913 | -0.430 | -0.7527 | Yes |
| 94 | HMGA1 |  |  | 1915 | -0.433 | -0.7509 | Yes |
| 95 | POLD3 |  |  | 1918 | -0.436 | -0.7495 | Yes |
| 96 | UHRF1 |  |  | 1921 | -0.438 | -0.7482 | Yes |
| 97 | DIAPH3 |  |  | 1924 | -0.439 | -0.7468 | Yes |
| 98 | KDELR2 |  |  | 1925 | -0.439 | -0.7444 | Yes |
| 99 | GGCT |  |  | 1930 | -0.442 | -0.7441 | Yes |
| 100 | GINS2 |  |  | 1932 | -0.445 | -0.7422 | Yes |
| 101 | SNRPB |  |  | 1937 | -0.447 | -0.7418 | Yes |
| 102 | MZT2B |  |  | 1941 | -0.449 | -0.7409 | Yes |
| 103 | TPRKB |  |  | 1944 | -0.450 | -0.7394 | Yes |
| 104 | CLIC1 |  |  | 1949 | -0.454 | -0.7390 | Yes |
| 105 | NUDC |  |  | 1950 | -0.454 | -0.7366 | Yes |
| 106 | RFC2 |  |  | 1954 | -0.457 | -0.7356 | Yes |
| 107 | DDX39A |  |  | 1957 | -0.458 | -0.7341 | Yes |
| 108 | CDT1 |  |  | 1961 | -0.460 | -0.7332 | Yes |
| 109 | ZWILCH |  |  | 1962 | -0.461 | -0.7307 | Yes |
| 110 | DNMT1 |  |  | 1969 | -0.465 | -0.7312 | Yes |
| 111 | GTF3A |  |  | 1972 | -0.467 | -0.7297 | Yes |
| 112 | TIMM10 |  |  | 1975 | -0.470 | -0.7281 | Yes |
| 113 | CMC2 |  |  | 1977 | -0.473 | -0.7261 | Yes |
| 114 | C19orf48 |  |  | 1979 | -0.474 | -0.7240 | Yes |
| 115 | POP7 |  |  | 1981 | -0.474 | -0.7220 | Yes |
| 116 | CSE1L |  |  | 1985 | -0.479 | -0.7209 | Yes |
| 117 | SUPT16H |  |  | 1986 | -0.479 | -0.7183 | Yes |
| 118 | TIMELESS |  |  | 1989 | -0.482 | -0.7167 | Yes |
| 119 | CDC6 |  |  | 1991 | -0.483 | -0.7146 | Yes |
| 120 | MCM4 |  |  | 1993 | -0.487 | -0.7125 | Yes |
| 121 | SKA1 |  |  | 1996 | -0.490 | -0.7108 | Yes |
| 122 | PSMC3 |  |  | 1999 | -0.492 | -0.7092 | Yes |
| 123 | FAM111A |  |  | 2002 | -0.497 | -0.7075 | Yes |
| 124 | TK1 |  |  | 2003 | -0.497 | -0.7048 | Yes |
| 125 | LRR1 |  |  | 2004 | -0.497 | -0.7021 | Yes |
| 126 | TUBB6 |  |  | 2006 | -0.500 | -0.6999 | Yes |
| 127 | CHEK1 |  |  | 2013 | -0.504 | -0.7002 | Yes |
| 128 | SIVA1 |  |  | 2014 | -0.505 | -0.6975 | Yes |
| 129 | MAGOHB |  |  | 2015 | -0.505 | -0.6947 | Yes |
| 130 | XRCC5 |  |  | 2016 | -0.507 | -0.6920 | Yes |
| 131 | HIRIP3 |  |  | 2017 | -0.508 | -0.6892 | Yes |
| 132 | FEN1 |  |  | 2018 | -0.511 | -0.6865 | Yes |
| 133 | SMC1A |  |  | 2021 | -0.513 | -0.6847 | Yes |
| 134 | RRM1 |  |  | 2023 | -0.516 | -0.6824 | Yes |
| 135 | RANBP1 |  |  | 2024 | -0.517 | -0.6796 | Yes |
| 136 | HAUS8 |  |  | 2026 | -0.517 | -0.6773 | Yes |
| 137 | CACYBP |  |  | 2028 | -0.518 | -0.6750 | Yes |
| 138 | NME4 |  |  | 2029 | -0.520 | -0.6722 | Yes |
| 139 | PARPBP |  |  | 2030 | -0.520 | -0.6694 | Yes |
| 140 | PDCD5 |  |  | 2031 | -0.520 | -0.6666 | Yes |
| 141 | DEPDC1B |  |  | 2037 | -0.527 | -0.6662 | Yes |
| 142 | HELLS |  |  | 2038 | -0.528 | -0.6634 | Yes |
| 143 | CDCA3 |  |  | 2041 | -0.529 | -0.6615 | Yes |
| 144 | CCT5 |  |  | 2042 | -0.529 | -0.6587 | Yes |
| 145 | TRIP13 |  |  | 2044 | -0.530 | -0.6563 | Yes |
| 146 | NCAPD3 |  |  | 2051 | -0.537 | -0.6564 | Yes |
| 147 | KIF22 |  |  | 2053 | -0.541 | -0.6540 | Yes |
| 148 | CENPJ |  |  | 2054 | -0.544 | -0.6511 | Yes |
| 149 | CKLF |  |  | 2055 | -0.544 | -0.6481 | Yes |
| 150 | SNRPF |  |  | 2057 | -0.545 | -0.6457 | Yes |
| 151 | CTNNAL1 |  |  | 2059 | -0.548 | -0.6432 | Yes |
| 152 | DTL |  |  | 2066 | -0.573 | -0.6432 | Yes |
| 153 | FANCD2 |  |  | 2068 | -0.577 | -0.6405 | Yes |
| 154 | ZWINT |  |  | 2069 | -0.577 | -0.6374 | Yes |
| 155 | CEP152 |  |  | 2070 | -0.578 | -0.6343 | Yes |
| 156 | RTKN2 |  |  | 2071 | -0.579 | -0.6311 | Yes |
| 157 | RACGAP1 |  |  | 2072 | -0.579 | -0.6280 | Yes |
| 158 | MND1 |  |  | 2074 | -0.586 | -0.6253 | Yes |
| 159 | GINS4 |  |  | 2075 | -0.586 | -0.6222 | Yes |
| 160 | LSM4 |  |  | 2076 | -0.587 | -0.6190 | Yes |
| 161 | SKA3 |  |  | 2077 | -0.590 | -0.6158 | Yes |
| 162 | BUB3 |  |  | 2078 | -0.596 | -0.6125 | Yes |
| 163 | AURKB |  |  | 2079 | -0.598 | -0.6093 | Yes |
| 164 | GGH |  |  | 2080 | -0.603 | -0.6060 | Yes |
| 165 | CENPK |  |  | 2082 | -0.607 | -0.6033 | Yes |
| 166 | USP1 |  |  | 2084 | -0.612 | -0.6004 | Yes |
| 167 | SSRP1 |  |  | 2091 | -0.630 | -0.6001 | Yes |
| 168 | MCM8 |  |  | 2092 | -0.635 | -0.5966 | Yes |
| 169 | FAM111B |  |  | 2094 | -0.637 | -0.5937 | Yes |
| 170 | MIS18A |  |  | 2096 | -0.639 | -0.5907 | Yes |
| 171 | DNAJC9 |  |  | 2097 | -0.641 | -0.5873 | Yes |
| 172 | RFC3 |  |  | 2098 | -0.642 | -0.5838 | Yes |
| 173 | BRCA2 |  |  | 2100 | -0.650 | -0.5808 | Yes |
| 174 | PHF19 |  |  | 2101 | -0.650 | -0.5772 | Yes |
| 175 | ITGB3BP |  |  | 2102 | -0.652 | -0.5737 | Yes |
| 176 | GNG4 |  |  | 2103 | -0.652 | -0.5702 | Yes |
| 177 | NCAPH |  |  | 2104 | -0.652 | -0.5666 | Yes |
| 178 | SNRPD1 |  |  | 2105 | -0.653 | -0.5631 | Yes |
| 179 | PSMC3IP |  |  | 2106 | -0.659 | -0.5595 | Yes |
| 180 | KPNB1 |  |  | 2108 | -0.660 | -0.5565 | Yes |
| 181 | CKAP2L |  |  | 2109 | -0.663 | -0.5529 | Yes |
| 182 | ALYREF |  |  | 2111 | -0.677 | -0.5497 | Yes |
| 183 | RPA3 |  |  | 2112 | -0.678 | -0.5460 | Yes |
| 184 | LSM3 |  |  | 2114 | -0.694 | -0.5428 | Yes |
| 185 | CKS2 |  |  | 2115 | -0.694 | -0.5390 | Yes |
| 186 | RAD21 |  |  | 2118 | -0.697 | -0.5363 | Yes |
| 187 | ANP32B |  |  | 2119 | -0.700 | -0.5325 | Yes |
| 188 | CDCA2 |  |  | 2120 | -0.705 | -0.5286 | Yes |
| 189 | PA2G4 |  |  | 2123 | -0.707 | -0.5258 | Yes |
| 190 | CENPM |  |  | 2125 | -0.709 | -0.5225 | Yes |
| 191 | HNRNPD |  |  | 2126 | -0.713 | -0.5186 | Yes |
| 192 | RAD51AP1 |  |  | 2127 | -0.715 | -0.5147 | Yes |
| 193 | HNRNPAB |  |  | 2130 | -0.729 | -0.5118 | Yes |
| 194 | CKAP2 |  |  | 2131 | -0.729 | -0.5079 | Yes |
| 195 | CIP2A |  |  | 2132 | -0.733 | -0.5039 | Yes |
| 196 | ATAD5 |  |  | 2133 | -0.742 | -0.4999 | Yes |
| 197 | VRK1 |  |  | 2134 | -0.743 | -0.4958 | Yes |
| 198 | TUBA1C |  |  | 2135 | -0.756 | -0.4917 | Yes |
| 199 | KIF2C |  |  | 2136 | -0.756 | -0.4876 | Yes |
| 200 | BRCA1 |  |  | 2138 | -0.758 | -0.4840 | Yes |
| 201 | CMSS1 |  |  | 2140 | -0.772 | -0.4804 | Yes |
| 202 | NDC80 |  |  | 2142 | -0.775 | -0.4767 | Yes |
| 203 | H2AFV |  |  | 2143 | -0.779 | -0.4725 | Yes |
| 204 | FBXO5 |  |  | 2144 | -0.779 | -0.4682 | Yes |
| 205 | KIF23 |  |  | 2145 | -0.782 | -0.4640 | Yes |
| 206 | GMNN |  |  | 2146 | -0.785 | -0.4597 | Yes |
| 207 | LMNB2 |  |  | 2147 | -0.786 | -0.4555 | Yes |
| 208 | MELK |  |  | 2148 | -0.786 | -0.4512 | Yes |
| 209 | SMC3 |  |  | 2150 | -0.800 | -0.4474 | Yes |
| 210 | KIFC1 |  |  | 2151 | -0.802 | -0.4430 | Yes |
| 211 | DEPDC1 |  |  | 2152 | -0.809 | -0.4387 | Yes |
| 212 | DEK |  |  | 2154 | -0.817 | -0.4347 | Yes |
| 213 | DUT |  |  | 2155 | -0.824 | -0.4303 | Yes |
| 214 | NCAPG2 |  |  | 2156 | -0.827 | -0.4258 | Yes |
| 215 | TUBB |  |  | 2157 | -0.831 | -0.4213 | Yes |
| 216 | MCM7 |  |  | 2160 | -0.839 | -0.4177 | Yes |
| 217 | TUBB4B |  |  | 2162 | -0.843 | -0.4137 | Yes |
| 218 | HMGB3 |  |  | 2164 | -0.852 | -0.4096 | Yes |
| 219 | SPC24 |  |  | 2166 | -0.853 | -0.4055 | Yes |
| 220 | ARHGAP11A |  |  | 2167 | -0.855 | -0.4008 | Yes |
| 221 | DTYMK |  |  | 2168 | -0.858 | -0.3962 | Yes |
| 222 | MCM10 |  |  | 2169 | -0.858 | -0.3915 | Yes |
| 223 | FOXM1 |  |  | 2171 | -0.861 | -0.3874 | Yes |
| 224 | CDCA5 |  |  | 2172 | -0.866 | -0.3827 | Yes |
| 225 | BUB1B |  |  | 2174 | -0.871 | -0.3785 | Yes |
| 226 | PIMREG |  |  | 2175 | -0.876 | -0.3737 | Yes |
| 227 | NUF2 |  |  | 2176 | -0.877 | -0.3690 | Yes |
| 228 | KPNA2 |  |  | 2178 | -0.894 | -0.3646 | Yes |
| 229 | CCDC34 |  |  | 2180 | -0.898 | -0.3603 | Yes |
| 230 | CCNA2 |  |  | 2181 | -0.902 | -0.3554 | Yes |
| 231 | CHAF1A |  |  | 2182 | -0.903 | -0.3505 | Yes |
| 232 | MIS18BP1 |  |  | 2183 | -0.903 | -0.3456 | Yes |
| 233 | SMS |  |  | 2184 | -0.910 | -0.3407 | Yes |
| 234 | KIF15 |  |  | 2185 | -0.915 | -0.3357 | Yes |
| 235 | KIF4A |  |  | 2186 | -0.921 | -0.3307 | Yes |
| 236 | ORC6 |  |  | 2188 | -0.937 | -0.3262 | Yes |
| 237 | C21orf58 |  |  | 2189 | -0.937 | -0.3211 | Yes |
| 238 | SGO1 |  |  | 2190 | -0.942 | -0.3160 | Yes |
| 239 | CENPH |  |  | 2191 | -0.943 | -0.3109 | Yes |
| 240 | CENPW |  |  | 2192 | -0.943 | -0.3057 | Yes |
| 241 | LMNB1 |  |  | 2194 | -0.950 | -0.3011 | Yes |
| 242 | HJURP |  |  | 2195 | -0.962 | -0.2959 | Yes |
| 243 | UBE2T |  |  | 2196 | -0.965 | -0.2907 | Yes |
| 244 | FANCI |  |  | 2198 | -0.966 | -0.2859 | Yes |
| 245 | CDKN3 |  |  | 2199 | -0.979 | -0.2806 | Yes |
| 246 | CKS1B |  |  | 2201 | -0.987 | -0.2758 | Yes |
| 247 | HIST1H1E |  |  | 2202 | -0.992 | -0.2704 | Yes |
| 248 | SGO2 |  |  | 2203 | -1.003 | -0.2650 | Yes |
| 249 | ESCO2 |  |  | 2204 | -1.004 | -0.2595 | Yes |
| 250 | SMC2 |  |  | 2205 | -1.006 | -0.2541 | Yes |
| 251 | EZH2 |  |  | 2207 | -1.017 | -0.2491 | Yes |
| 252 | SPC25 |  |  | 2208 | -1.019 | -0.2435 | Yes |
| 253 | XRCC2 |  |  | 2210 | -1.028 | -0.2385 | Yes |
| 254 | DHFR |  |  | 2212 | -1.040 | -0.2333 | Yes |
| 255 | KNL1 |  |  | 2213 | -1.054 | -0.2276 | Yes |
| 256 | NUDT1 |  |  | 2214 | -1.054 | -0.2219 | Yes |
| 257 | MYBL2 |  |  | 2215 | -1.058 | -0.2162 | Yes |
| 258 | CLSPN |  |  | 2216 | -1.060 | -0.2104 | Yes |
| 259 | CENPN |  |  | 2217 | -1.079 | -0.2046 | Yes |
| 260 | UBE2C |  |  | 2218 | -1.081 | -0.1987 | Yes |
| 261 | CENPU |  |  | 2219 | -1.088 | -0.1928 | Yes |
| 262 | H2AFZ |  |  | 2220 | -1.090 | -0.1869 | Yes |
| 263 | NCAPG |  |  | 2222 | -1.122 | -0.1813 | Yes |
| 264 | H2AFX |  |  | 2223 | -1.146 | -0.1751 | Yes |
| 265 | CDK1 |  |  | 2225 | -1.153 | -0.1694 | Yes |
| 266 | ANP32E |  |  | 2227 | -1.200 | -0.1634 | Yes |
| 267 | MAD2L1 |  |  | 2228 | -1.204 | -0.1569 | Yes |
| 268 | GTSE1 |  |  | 2230 | -1.220 | -0.1508 | Yes |
| 269 | DBF4 |  |  | 2232 | -1.247 | -0.1445 | Yes |
| 270 | RRM2 |  |  | 2234 | -1.268 | -0.1382 | Yes |
| 271 | SMC4 |  |  | 2236 | -1.281 | -0.1317 | Yes |
| 272 | PBK |  |  | 2237 | -1.325 | -0.1245 | Yes |
| 273 | ATAD2 |  |  | 2238 | -1.381 | -0.1170 | Yes |
| 274 | TMPO |  |  | 2239 | -1.382 | -0.1096 | Yes |
| 275 | PRC1 |  |  | 2240 | -1.397 | -0.1020 | Yes |
| 276 | HMGB2 |  |  | 2241 | -1.469 | -0.0940 | Yes |
| 277 | BIRC5 |  |  | 2242 | -1.469 | -0.0861 | Yes |
| 278 | ASPM |  |  | 2244 | -1.490 | -0.0785 | Yes |
| 279 | PCLAF |  |  | 2245 | -1.516 | -0.0703 | Yes |
| 280 | TOP2A |  |  | 2246 | -1.562 | -0.0618 | Yes |
| 281 | NUSAP1 |  |  | 2247 | -1.600 | -0.0531 | Yes |
| 282 | TPX2 |  |  | 2248 | -1.609 | -0.0444 | Yes |
| 283 | CENPF |  |  | 2249 | -1.691 | -0.0352 | Yes |
| 284 | HIST1H1D |  |  | 2250 | -1.720 | -0.0259 | Yes |
| 285 | MKI67 |  |  | 2252 | -1.892 | -0.0162 | Yes |
| 286 | HIST1H4C |  |  | 2253 | -2.984 | 0.0000 | Yes |
Table: GSEA details [plain text format]

  

Fig 2: DOROSZKO\_U3180MG\_CLUSTER\_3: Random ES distribution      
 Gene set null distribution of ES for **DOROSZKO\_U3180MG\_CLUSTER\_3**
